# Detection of *Alu* Exonization Events in Human Frontal Cortex From RNA-Seq Data

**DOI:** 10.3389/fmolb.2021.727537

**Published:** 2021-09-10

**Authors:** Liliana Florea, Lindsay Payer, Corina Antonescu, Guangyu Yang, Kathleen Burns

**Affiliations:** ^1^ McKusick-Nathans Department of Genetic Medicine, Johns Hopkins School of Medicine, Baltimore, MD, United States; ^2^ Department of Computer Science, Johns Hopkins University, Baltimore, MD, United States; ^3^ Department of Pathology, Johns Hopkins School of Medicine, Baltimore, MD, United States; ^4^ Department of Pathology, Dana-Farber Cancer Institute, Boston, MA, United States; ^5^ Harvard Medical School, Boston, MA, United States

**Keywords:** *Alu* exonization, alternative splicing, RNA sequencing, computational prediction, frontal cortex

## Abstract

*Alu* exonization events functionally diversify the transcriptome, creating alternative mRNA isoforms and accounting for an estimated 5% of the alternatively spliced (skipped) exons in the human genome. We developed computational methods, implemented into a software called Alubaster, for detecting incorporation of *Alu* sequences in mRNA transcripts from large scale RNA-seq data sets. The approach detects *Alu* sequences derived from both fixed and polymorphic *Alu* elements, including *Alu* insertions missing from the reference genome. We applied our methods to 117 GTEx human frontal cortex samples to build and characterize a collection of *Alu*-containing mRNAs. In particular, we detected and characterized *Alu* exonizations occurring at 870 fixed *Alu* loci, of which 237 were novel, as well as hundreds of putative events involving *Alu* elements that are polymorphic variants or rare alleles not present in the reference genome. These methods and annotations represent a unique and valuable resource that can be used to understand the characteristics of *Alu*-containing mRNAs and their tissue-specific expression patterns.

## Introduction

*Alu* elements are ∼300 bp sequences belonging to an order of retrotransposons termed Short Interspersed Elements (SINEs) that have expanded in primates (Batzer and Deininger, 2002; Hormozdiari et al., 2013). *Alu* elements represent 11% of the human genome, with nearly one million copies located primarily in introns and intergenic space proximal to genes (Lander et al., 2001; Venter et al., 2001). They have contributed to genetic and functional diversity during evolution in multiple ways. *Alu* element insertions can influence gene regulation and affect RNA polyadenylation, splicing, and editing (Chen et al., 2008; Chen et al., 2009; Vorechovsky, 2010; Deininger, 2011; Shen et al., 2011; Payer and Burns, 2019). *Alu* elements can be deleterious, interrupting key gene regulatory elements, and serving as substrates for non-allelic recombination leading to copy number variations (CNVs).

*Alu* exonization, or recruitment of an intronic *Alu* element into a gene transcript can alter protein sequence (Lev-Maor et al., 2003) and function or, alternatively, can introduce a premature termination codon (PTC) and trigger nonsense mediated decay (Attig et al., 2016) (NMD) surveillance mechanisms to degrade transcripts. Examples of *Alu* exonizations causing disease include a mutation in an antisense *Alu* element, which created a splice donor and activated a cryptic splice acceptor in the ornithine-delta-aminotransferase mRNA leading to a loss-of-function (Mitchell et al., 1991). Other examples of mutations that lead to *Alu* exonization include a collagen, type IV, alpha 3 (Goodpasture antigen) (*COL4A3*) allele causing Alport syndrome (Knebelmann et al., 1995); a survivin allele (baculoviral IAP repeat containing 5 (*BIRC5*)) causing Sly syndrome (Mola et al., 2007); a fibroblast growth factor receptor 2 (*FGFR2*) allele causing Apert syndrome (Oldridge et al., 1999); and a 6-pyruvoyltetrahydropterin synthase (*PTS*) allele resulting in tetrahydrobiopterin deficiency (Meili et al., 2009).

*Alu* elements have contributed directly and in a significant way to the creation of new gene content. Approximately 5% of alternatively spliced exons internal to the human genes are estimated to have derived from exonizations of intronic *Alu* sequences (Sorek et al., 2002). The most common mechanism is *via* changes in the *Alu* sequence leading to the formation of new and typically weak 5′ splice sites (ss) (Lev-Maor et al., 2003). There are multiple potential 5’ ss, and the selection of a specific site is determined by a complex interplay between the relative strength of the candidate splice sites, coupled with splicing regulatory elements (enhancers) within the *Alu* exon sequence (Ram et al., 2008). There is a proclivity for *Alu* exonization when an intronic *Alu* is oriented antisense to the gene (Lev-Maor et al., 2003; Sela et al., 2007; Vorechovsky, 2010). The primary path to exonization is *via* alternative splicing, with the transcript incorporating the new *Alu* exon starting off as the minor isoform. From an evolutionary perspective, this scenario may allow a locus to “experiment” with new function while preserving its primary function. Over time, mutations in the *Alu* exon and surrounding intron sequence, and the selective pressures acting on them, may lead to relative permanence or the acquisition of new function, and to the transcript being promoted to the major isoform. Indeed, a significant number of the exonized *Alu* elements are unique to the human genome, and similarly for other primates, and thought to have played a part in the formation of species-specific traits (Shen et al., 2011).

With the ascent and democratization of deep RNA sequencing (RNA-seq), there are now vast collections of detailed gene expression data from large numbers of individuals, species and developmental or cellular conditions. Such resources present tremendous opportunities to identify instances of *Alu* element recruitment into transcripts under a wide variety of conditions. In particular, cataloguing exonization of fixed *Alu* elements may increase our understanding of tissue, organ and cell type specificity, and help uncover niche functions evolved through *Alu* exonizations. Such a wholesale discovery and curation effort has not yet been undertaken for *Alu* exons, and they remain poorly represented in the gene annotation databases.

While significantly improved in its representation of the *Alu* content and haplotype variation, the current reference genome is incomplete as a representation of individuals. *Alu* elements continue to insert into the human genome and to create structural variants, at a rate of about one new *Alu* insert per 20 human births (Xing et al., 2009; Deininger, 2011). This process leads to genetic diversity (Comas et al., 2004), but also to about one in 1,000 new human genetic (Deininger and Batzer, 1999; Vorechovsky, 2010) diseases. Understanding the preponderance and impact of polymorphic and rare *Alu* insertion variants in the population and in disease is a yet untapped reservoir.

Detection of *Alu* exonization from short read, high-throughput RNA-seq data, however, is challenging for multiple reasons. When the *Alu* is incorporated in the reference genome, *Alu*-containing RNA-seq reads may map to multiple locations on the genome or even within the same gene locus, leading to their exclusion or making it difficult to unambiguously determine the source. Further, unspliced, pre-mRNA sequences are present in RNA-seq experiments (Ameur et al., 2011) and *Alu*-containing reads from pre-mRNA can be difficult to distinguish from fully processed mRNAs containing *Alu-*derived exons. Furthermore, when the *Alu* element is not part of the reference assembly (i.e., is a polymorphic or *de novo* element), the location of the insert in the genome may be unknown. Most of these *Alu* insertions are members of a subfamily of *Alu*Y elements (Batzer and Deininger, 1991). The *Alu*Y subfamily is one of several that account for almost all recently integrated human *Alu* elements, including rare and polymorphic events not represented in the reference human genome (Batzer and Deininger, 1991; Batzer and Deininger, 2002). High similarity among *Alu*Y elements coupled with sequencing errors make it difficult to distinguish reads as derived from a non-reference *Alu* as opposed to from another *Alu*Y residing in the same intron. Adding to the complexity, unlike with DNA sequences where *Alu* insertions can be detected as local structural variations (Qian et al., 2015) that appear as breaks in the expected co-localization of paired end reads, the interrupted structure of mRNAs allows for reads in the same pair to be arbitrarily distanced, increasing the likelihood of a local *Alu* element (‘shadow’) confounding the prediction and creating a false positive. The precise site of a genomic *Alu* may be impossible to pinpoint even when an exonization event is evident. Lastly, most of these elements may be expressed at very low levels, limiting their deleterious effects but providing little read evidence to allow detection. The challenges inherent to identifying good candidate sites and distinguishing these from a large potential number of false signals make the task of predicting novel *Alu* exonizations particularly daunting.

We describe two methods for detecting *Alu* insertions in mRNA sequences, at elements already included in the reference genome, and at novel loci not encoded in the reference genome and representing likely polymorphic or rare variations, respectively. We then apply the tools to a collection of 117 RNA-seq data sets from human frontal cortex tissue, generated by the GTEx project (Consortium, 2015). The collection of sequences and annotations can be used as a starting point for validation experiments, and incorporated into functional and disease studies targeted at the feature level. Lastly, our study is a model for creating a comprehensive *Alu* mRNA feature repertoire in other tissues, across developmental stages and for a wide variety of disease and normal cellular conditions.

We implemented the algorithm into a software called Alubaster, available from https://github.com/splicebox/Alubaster.

## Results

### Detection of Exonization Events at *Alu* Elements in the Reference Genome

When the DNA sequence of an *Alu* insertion variant is included in the reference genome, traditional approaches to read alignment and RNA-seq analysis can be used to distinguish between *Alu* exonization (‘signal’) and ‘noise’ generated by unprocessed intronic RNA and from multi-mappings reads. Transcript assembly algorithms incorporate intronic read filters, using statistical modeling to distinguish intronic read levels in mRNA resulted from intron retention and alternatively spliced exons, including exonized *Alu* elements, and unprocessed RNA from incomplete co-transcriptional splicing (Song et al., 2016). Additionally, assemblers can detect and remove splicing patterns that have very low likelihood, for instance with low read support or creating uncharacteristic mRNA features, such as unexpectedly long introns and exons. Further, rather than assessing each read to determine its origin among multiple mappings on the genome, assemblers collectively analyze clusters of reads to assess the relative contribution of unique and multi-mapping sequences at the same location and to filter out potential paralogs.

**FIGURE 1:**
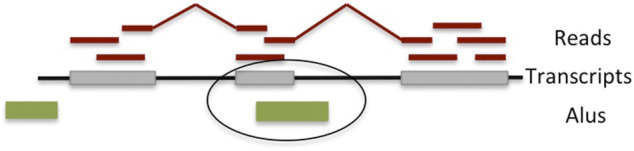
Overview of the detection algorithm for fixed (in the reference genome) *Alu* exonizations. RNA-seq reads are mapped to the reference genome and assembled into transcripts, some of which contain the *Alu* exons. Exons overlapping an *Alu* element located on the opposite strand and that are between 40 and 400 bp long are deemed to have occurred through *Alu* exonization.

We used the transcript assembler CLASS2, which implements all of the above filters and has been shown to capture alternative splicing variation, especially within the body of the gene, with very high accuracy (Song et al., 2016). Candidate *Alu*-containing exons that are internal to a gene, agree in size (<400), and are in antisense orientation to the annotated *Alu* are further retained (Figure 1). The exons are then collected across all samples to create a comprehensive list.

### *Alu* Exonization Events in the Human Frontal Cortex

**FIGURE 2:**
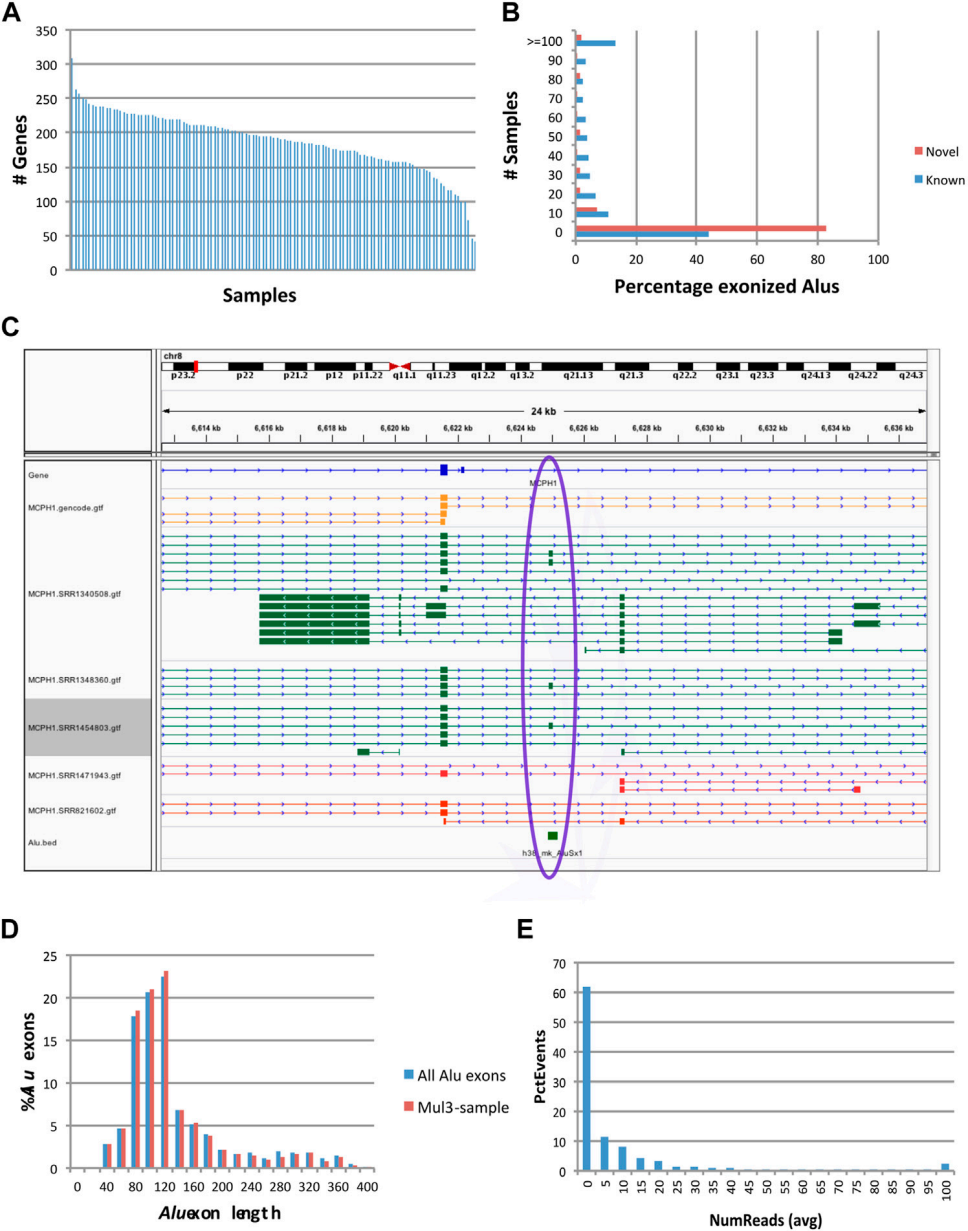
Characteristics of exonization events at fixed (reference) *Alu* loci. Events occurred at 870 reference *Alu* loci, of which 633 occurred at GENCODE annotated exons (‘known’) and 237 had not been previously known (‘novel’). **(A)** Numbers of genes with *Alu* exonization events, by sample. **(B)** Prevalence of *Alu* exonization events in the 117 samples. **(C)** Example of a fixed *Alu*Sx exonization event at the microcephalin 1 (MCPH1) gene. Gold: GENCODE gene annotations. Green and Red: samples with and without evidence of *Alu* exonization, respectively. This visualization was generated using the Integrated Genomics Viewer (IGV) (Robinson et al., 2011). **(D)** Histogram of *Alu* exon lengths. **(E)** Read counts of *Alu* exonization events. Total numbers of reads supporting the two flanking exons of the *Alu* exon were computed per sample, then averaged across samples and plotted into a histogram.

We analyzed 117 human frontal cortex RNA-seq samples obtained from the GTEx (Consortium, 2015) repository, with 16,495,334-172,877,906 reads per sample (92,601,615 sample average). When assembled, the reads produced between 26,164 and 66,638 transcripts per sample. In the first pass, applying the exon filters above detected 1,019 *Alu* exons (45-343 per sample), at 870 reference *Alu* elements (loci) in 861 genes (Figure 2A and Supplementary Table S1). Of these, 725 events were found in 2 or more samples. Because the transcript assembly process may be too stringent to capture low expression exons, including some *Alu* exons, we relax the criteria to allow counting an exon in a sample if its flanking introns have read support. After expanding the search, 947 *Alu* exons, representing 92.9% of the total, had concomitant evidence for both splice sites in more than 2 samples. Therefore, the collection of *Alu* exonization events generated with our assembly-based method appeared robust and reliable.

*Alu* exonization events occurred across multiple subfamilies of *Alu* (467 *Alu*J, 357 *Alu*S, 40 *Alu*Y and 6 generic *Alu* instances). 633 of the exonized *Alu* elements were already annotated as exons in the GENCODE v.36 reference database, whereas 237 were not known to be exonized. A larger proportion of *Alu* elements known to undergo exonization had support in multiple samples compared to novel (unannotated) exonization events (Figure 2B). An example of a previously uncharacterized *Alu* exonization event at the *Microcephalin 1* (MCPH1) gene is shown in Figure 2C, in which the event presents as an alternatively spliced 142 bp exon (chr8:6624896-6625037). The 142 exon inserts between exon 13 and the 3’ terminal exon 14, introducing a frame shift in the coding sequence of ENST00000344683.8 and creating a longer reading frame (from 2,508 bp/836 aa to 2,628 bp/875 aa), by reading through the new exon and 11 new aa into the terminal exon. While the new *Alu* exon is included in transcript predictions from 3 samples (SRR1340508, SRR1454803 and SRR1348360), the upstream and downstream flanking introns are present at very low levels (<3 reads) in 67 samples and 7 samples, respectively, suggesting that the exon may be expressed more broadly.

### Features of *Alu* Exonization Events

*Alu* exon lengths were preponderantly between 80 and 140 bp (61.1%), with an average length of 144 bp and median 122 bp (Figure 2D). They were relatively uniformly distributed among in frame, frame +1 and frame +2 (342, 354, 323), and the distribution did not change when only the 947 exons present in multiple samples were considered (321, 323, 303). The above length distribution suggests that a majority of these *Alu* exonizations would likely be deleterious in a coding context, and hence may occur in non-coding RNAs or in untranslated regions of coding transcripts, or may be expressed at a low level that does not affect the overall output of the gene. Indeed, most events appear to show low read support in samples (Figure 2E).

### *Alu* Exonization Events Are Alternatively Spliced

The primary mechanism for *Alu* inclusion into mRNA structure is *via* alternative splicing. We compared the exon-intron structure of the predicted transcripts across the 117 samples to determine alternative splicing events involving our *Alu* exons, especially cassette (skipped) exons. We imposed a stringent requirement to only consider simple events, where the exon had to be excised or included into the transcript without any changes to the flanking exons, to isolate the effects of the exon sequence from those of the context. 651 (63.9%) of *Alu* exons were found to undergo exon skipping, in 785 alternative splicing patterns (ASP) formed by alternative flanking introns (Supplementary Table S2).

We further analyzed the expression level of the 785 ASPs across all samples, as reflected by the read coverage, and the relative isoform expression levels. The Percent Splice In (PSI) value is defined as the ratio of expression levels of the exon-including isoform and the isoforms containing the locus, and is used to measure the relative isoforms’ contribution (Wang et al., 2008). After correcting for samples with low numbers of reads (< 10), which could not render a reliable PSI estimate, 68.6% of the remaining 303 events had the *Alu* exon included in the minor isoform (PSI<0.35), 11.2% had relatively equal contributions of the two forms (0.36<=PSI<0.65), and in 20.1% of ASPs the exon was included in the major isoform (PSI>=0.65) (Supplementary Figure S1 and Supplementary Table S2). This distribution is in sharp contrast to that observed when all of the exon skipping events in the 117 samples data set were considered (39,859 events), where the vast majority of the skipped exons, 77.9%, were expressed as part of the major isoform (PSI>=0.65).

### Tissue Specificity of *Alu* Exonization Events

Lastly, we assessed the tissue specificity of 2,771 introns flanking the *Alu* exons by interrogating a comprehensive human introns database with the tool Snaptron (Wilks et al., 2018). Snaptron uses a collection of exon-exon junctions (introns), along with supporting read counts per sample, extracted from ∼50,000 publicly available RNA-seq samples, including those from the GTEx project. Read support for the 2,771 introns was extracted for all RNA-seq samples represented in GTEx, and a custom statistical test was designed to assess tissue specificity (see **Methods**). A total of 2,745 introns were found by Snaptron (Wilks et al., 2018), of which 45 introns were detected as brain specific using our stringent criteria (min 10 reads per sample, p-val<0.001). Roughly half of these introns occur in long non-coding RNAs (Supplementary Table S3).

**FIGURE 3:**
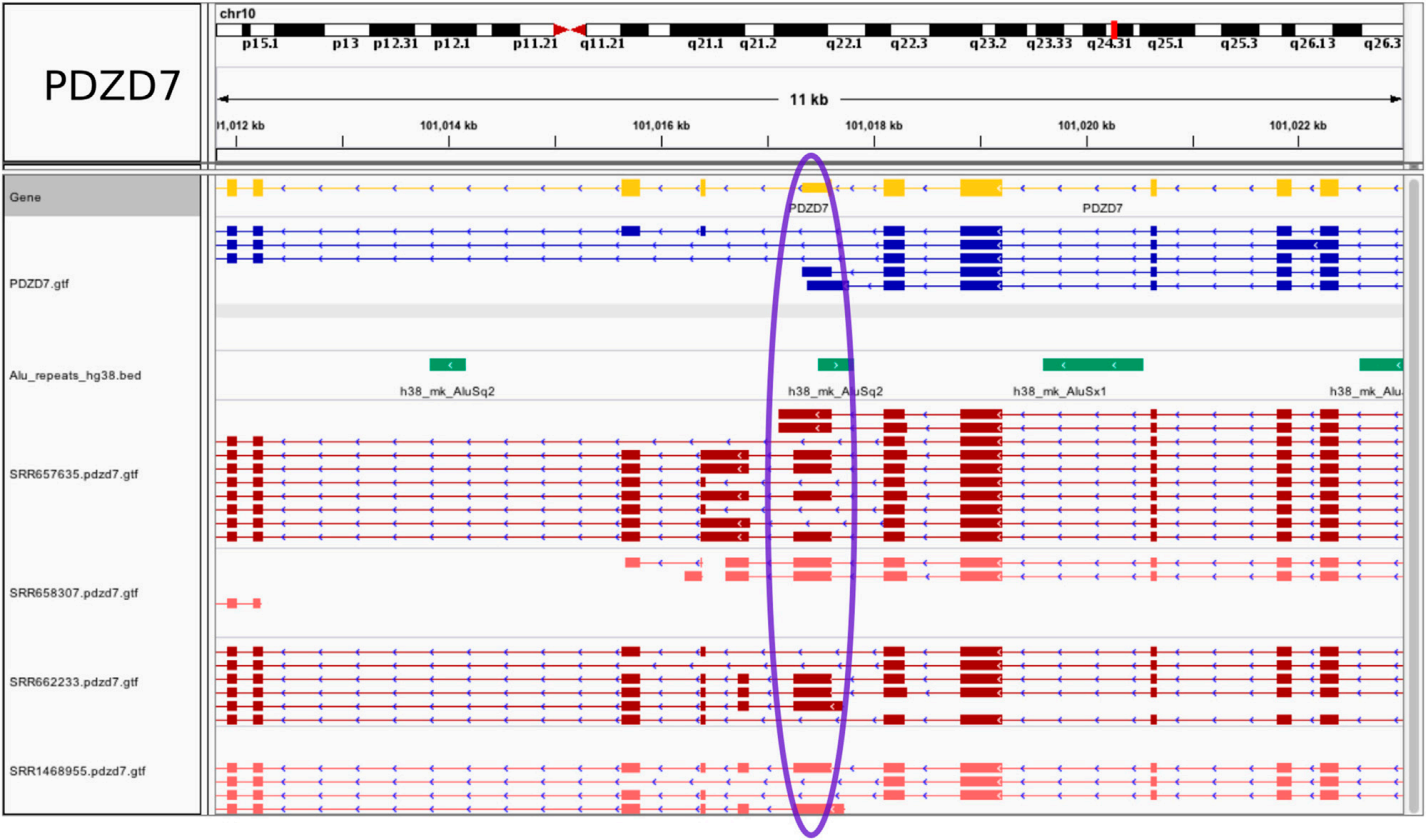
Fixed *Alu* exonization event in human frontal cortex at the PDZD7 gene. A gain of 5’ss at a 3′ UTR terminal exon at the PDZD7 gene creates a 363 bp internal *Alu* exon. The exon region is marked in blue on the display. The visualization was generated using the Integrated Genomics Viewer.

Examples of tissue specific events are illustrated in Figure 3 and Supplementary Figure S2. All (coding) host genes were confirmed to be expressed solely in brain using the ProteinAtlas resource (https://www.proteinatlas.org/), except for Ubiquitin-Associated Protein 1-Like (UBAP1L), also expressed in retina, which nevertheless leaves the possibility that the *Alu* exonization may take place preferentially in brain. At the PDZ Domain Containing 7 (PDZD7) gene, an annotated *Alu*-containing terminal 3′ exon is converted into an internal *Alu* exon (chr10:101017253-101017615) with the introduction of a novel, not present in the GENCODE annotation, brain specific intron, chr10:101016831,101017253. The resulting exon is 363 bp long. Additional novel exons and splicing patterns were also revealed by the RNA-seq data (Figure 3). In another example, transcripts assembled for the gene UBAP1L show a partially exonized *Alu*Sc element, to generate a 118 bp exon (chr15:65107209-65107326) not present in the GENCODE annotation, along with an additional potential *Alu* recruitment event within the upstream exon (Supplementary Figure S2A). The downstream intron, chr15:65106359,65107209 is tissue specific and not found in the reference database. Further, exonization of an *Alu*Jo element creates a new exon chr11:45528970-45529061 within the long non-coding RNA AC103855.2. Here, both of the flanking introns (chr11:45528443,45528970 and chr11:45529061,45531525) were identified as tissue specific and are not annotated in GENCODE. The combination of new exons and new complex splicing patterns internal to and/or at the 3’ end of the gene suggest the presence of multiple previously unknown splice isoforms that may perform brain specific functions (Supplementary Figure S2B). Lastly, and serving as a control, an annotated *Alu* exon (chr5:162102554-162102673) at the GABRG2 gene is flanked by the brain specific intron chr5:162101317,162102554 (Supplementary Figure S2C). As expected, in all of these cases the exonized *Alu* is present uniquely in primates, as shown in the UCSC Genome Browser plots.

### Prediction of Exons From Novel (Non-reference) *Alu* Insertions

When the DNA sequence of an *Alu* insertion variant is *not* part of the reference genome, standard RNA-seq analysis will discard or misplace informative reads. However, if reads or read pairs exist that span portions of the *Alu* element and of the adjacent exons, the unique exonic sequences flanking the exonization event can be used to ‘anchor’ the insertion and narrow down candidate reads for assembly.

**FIGURE 4:**
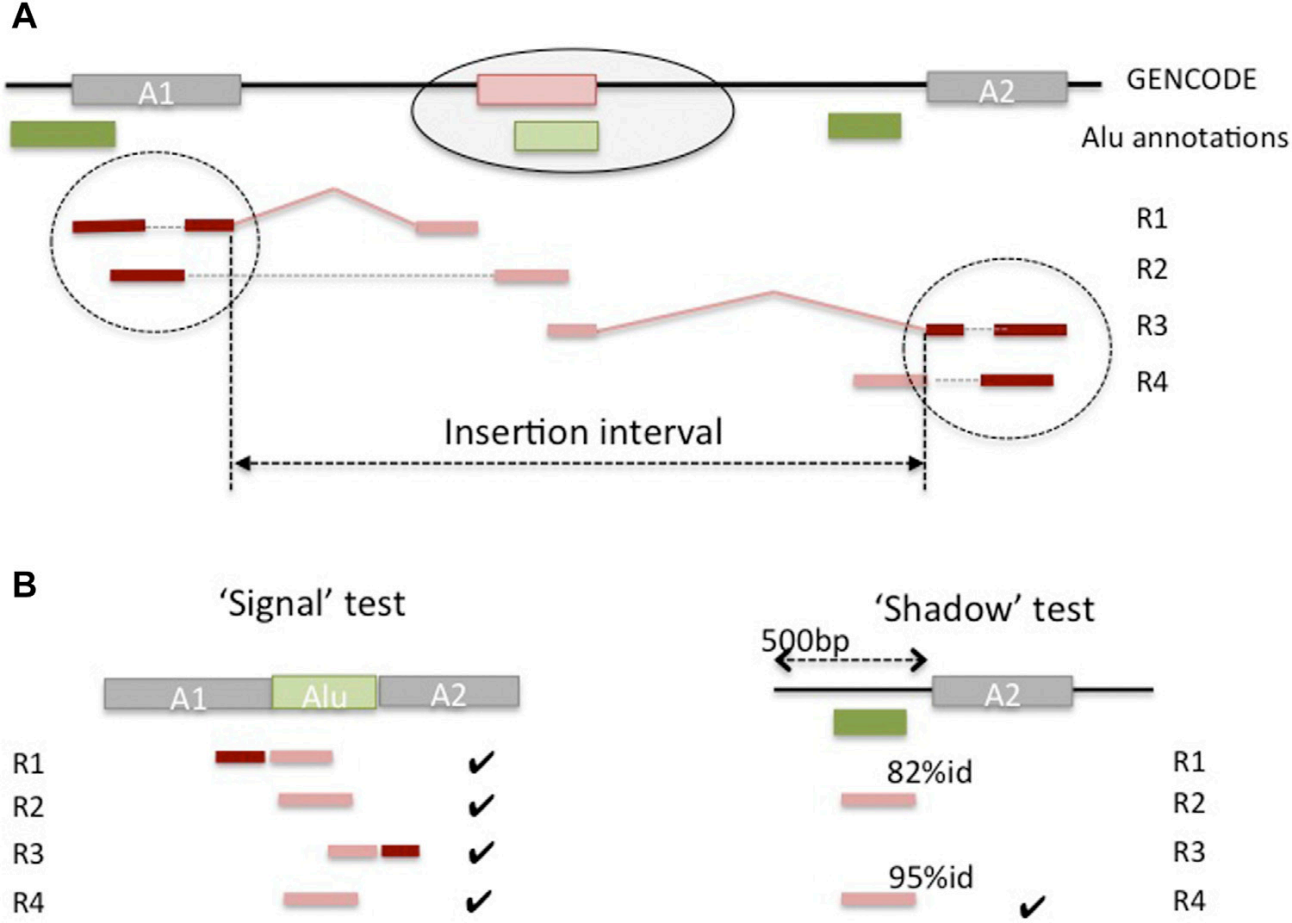
Outline of prediction of non-reference *Alu* insertions in genes. **(A)** A novel *Alu* insertion (light green) in a gene creates a novel exon (pink) that is not present in the genome, and for which there is evidence from RNA-seq reads (R1, R2, R3 and R4). Read pairs R1, R4 are all non-concordant and illustrate different scenarios. R1 has the first read mapped to (unique) sequence in anchor exon A1, and its mate is spliced between A1 and the new exon and missed by the aligner; similarly for R3, at the anchor exon A2. R2 has the first read mapping to A1, and its mate maps entirely inside the new exon. R4 shows a case of a ‘shadow’ read, from unprocessed intronic RNA, and was discarded by the aligner because it exceeded the maximum number of hits on the genome (e.g., 10 hits). **(B)** The ‘signal’ test matches the unmapped *Alu*-containing mate against the concatenation of the two anchor exons and the consensus *Alu* sequence. The ‘shadow’ test searches the same mate against the sequence adjacent to the anchor exon (shown), and against the genomic interval spanning the anchor exon and its neighbor (not shown).

We developed a pipeline to detect candidate *Alu* insertion loci in the genes, either within existing exons or as novel exons created by *Alu* exonizations. Given the interrupted structure of the gene and RNA-seq read alignments, it is not possible to *a priori* distinguish between these two cases; rather, the length of the predicted insertion interval can be used to infer the type of event from the predictions. To identify candidate insertion loci, we located mapped reads (‘anchors’) whose mates in the read pair contain a portion of an *Alu* sequence and could not be mapped on the genome (‘floats’) (Figure 4). To reduce the potentially large number of spurious matches, we only considered anchor reads overlapping annotated exons and therefore associated with known genes. A candidate read must then show evidence that it has originated from an exonized *Alu* sequence (‘signal’ test) and that is not likely to be sourced from a local reference *Alu* (‘shadow’ test). More specifically, a read is labeled as a ‘signal’ if it matches both a portion of the adjacent exonic sequences and a portion of the consensus *Alu* sequence. A read is deemed to be a ‘shadow’ if it aligns nearly exactly to the genomic sequence of a known *Alu* at the locus (see **Methods**). Further, to allow for small inaccuracies in the classification, the relative proportion of ‘signal’ to ‘shadow’ matches for a candidate insertion locus is used to call a putative novel *Alu* insertion event. Lastly, anchor reads and their ‘floats’ at that locus are assembled using a transcript assembly algorithm, which allows for multiple assembled sequences, potentially corresponding to different haplotypes. The assembled contigs are finally searched against the reference DNA sequence to eliminate any false positives missed in the previous steps, and to select a high confidence set of inserts for future curation and validation studies. The algorithm and calibration are described in detail in the **Methods**.

### Non-Reference *Alu* Insertion Events in the Human Frontal Cortex

**FIGURE 5:**
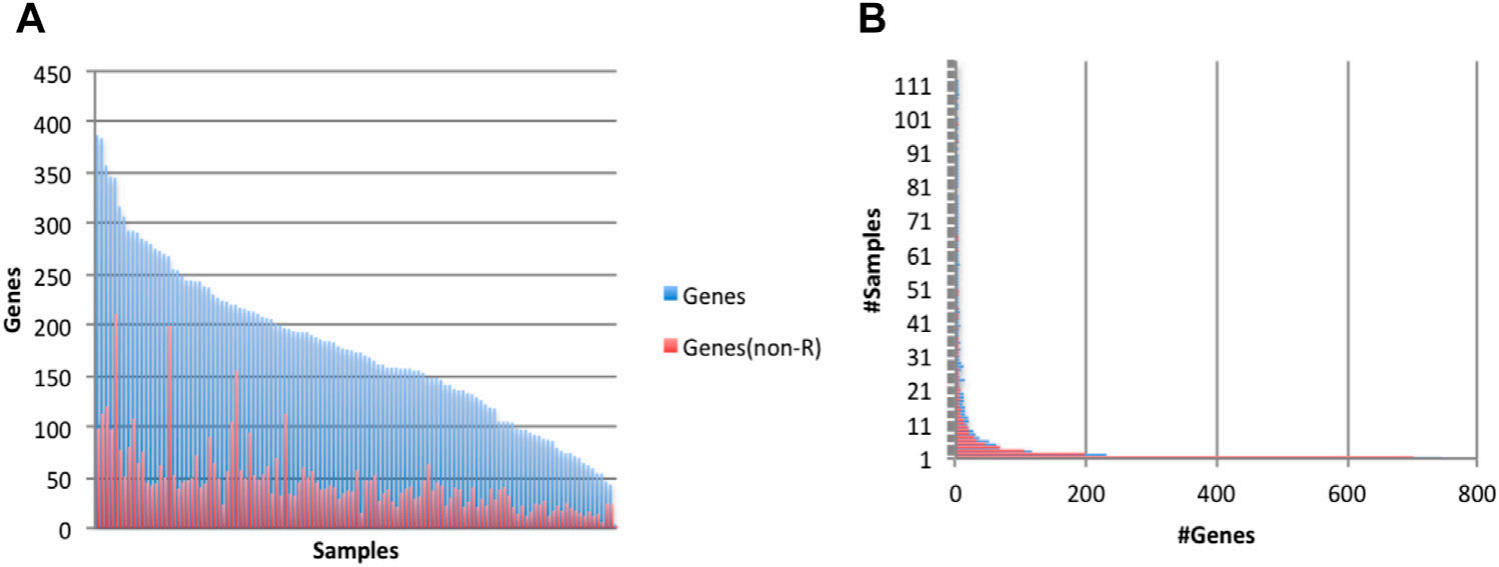
Characteristics of predicted (non-reference) *Alu* insertion events. Events were predicted to occur in 1,816 gene (1,353 genes when considering only events in a non-repeat rich context, ‘non-R’). **(A)** Numbers of genes (total and ‘non-R’) with putative *Alu* insertion events, by sample. **(B)** Prevalence of *Alu*-acquiring genes in the 117 samples.

We analyzed the 117 human frontal cortex samples above to determine candidate sites of *Alu* insertions. We detected putative polymorphic *Alu* insertions in 1,816 genes (4-387 per sample), of which 1,070 genes were reported to have undergone *Alu* insertions in multiple samples (Figure 5A). Many of the predicted *Alu* insertions were detected in repeat-rich regions, which makes them difficult to analyze and curate. Events at 1,353 genes (651 genes reported in multiple samples) occurred in non-repeat rich context (Figure 5B). The assembly process generated 11-1,756 contigs per sample; 17,585 contigs across all samples passed the alignment filter, at 1,363 genes.

**FIGURE 6:**
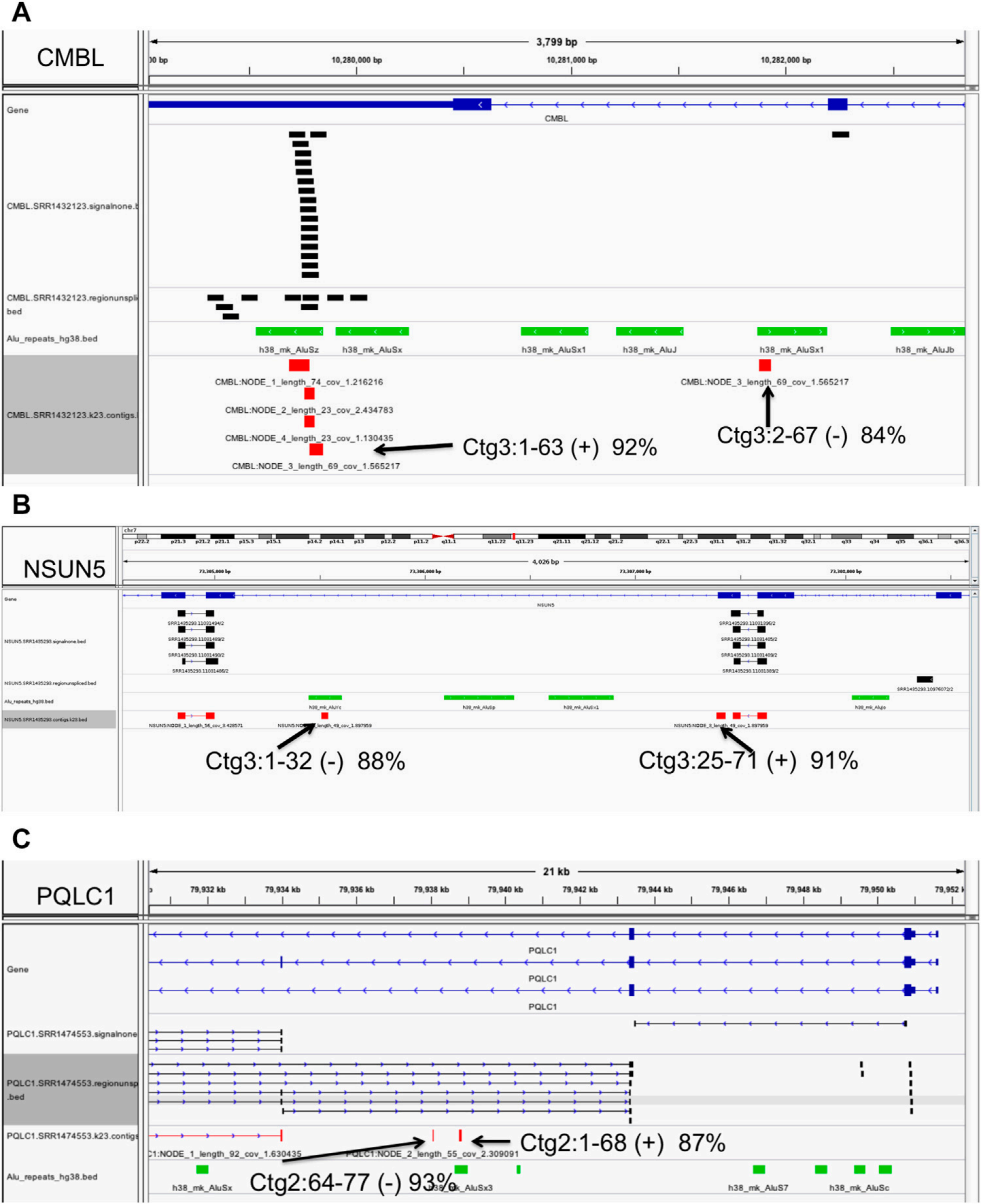
Example of polymorphic (non-reference) *Alu* insertions. Predicted *Alu* element insertions at the CMBL **(A)**, NSUN5 **(B)** and PQLC1 **(C)** genes. Annotations: black - clusters of ‘signalnone’ and ‘regionunspliced’ matches flanking the insertion site (top), green - local *Alu* elements, and red - alignments of assembled contigs. Full descriptions of the predicted *Alu* insertion events are presented in the text and **Methods**. Displays were generated with the Integrative Genomics Viewer.

We present several examples in Figure 6. For instance, at the Carboxymethylenebutenolidase-Like (*Pseudomonas*) (CMBL) gene, our algorithm predicts an *Alu* insertion within the interval chr5:10279825-10282219 in the gene’s 3′ most intron, in one sample only, SRR1432123 (Figure 6A). Of the four contigs assembled, three have high-quality (> 95% sequence identity) matches to the region, covering the full length of the contig sequences. The fourth, a 91 bp contig, has only partial paralogous matches to two separate local *Alu*Sz and *Alu*Sx1 sequences, at low 88 and 92% identity. Therefore, contig three is a likely novel *Alu* insertion. In a second example at the NOP2/Sun RNA methyltransferase 5 (NSUN5) gene (Figure 6B), the algorithm predicts an *Alu* exonization between positions 73,305,031 and 73,307,446 on chromosome 7, in 39 samples. In the sample SRR1435293, the 71 bp contig 3 has a high quality (91% sequence identity) partial match to an anchor exon, covering positions 25-71. The first 32 bases match in reverse orientation to a local intronic *Alu*, at 88% sequence identity, indicating a paralogous match. Therefore, contig three presents evidence of the insertion sequence spanning the junction between the *Alu* exon and the downstream anchor exon. Lastly, a novel *Alu* exonization is predicted at the PQ Loop Repeat Containing one/Solute Carrier Family 66 Member 2 (PQLC1/SLC66A2) gene locus, between 79,934,021 and 79,943,460 on chromosome 18. In sample SRR1474553, bases 1-68 of the 77 bp contig two have a low 87% identity match to the local *Alu* element which appears paralogous, and therefore is likely to represent a portion of the novel *Alu* insertion (Figure 6C).

## Methods

### Detection of Fixed *Alu* Exonization

When an exonization event occurs at an *Alu* annotated in the genome, standard RNA-seq analysis approaches can be used to identify the isoforms containing it. Each of the 117 samples were analyzed as follows. RNA-seq reads were assessed for quality and mapped to the GRCh38 genome (chromosomes 1-22, X, Y and M; https://ensembl.org/Homo_sapiens/) with the program TopHat2 v.2.1.1 (Kim et al., 2013). Reads were then assembled into transcripts with the transcript assembler CLASS2 v.2.1.6 with the sensitive setting, ‘–f 0.01’. With this option, CLASS2 reports all isoforms that are present at 1% or more of the expression level of the most abundant isoform for that gene. CLASS2 with the ‘-f 0.01’ option was previously shown to detect more splicing variation, in particular events at internal exons such as exon skipping, and more accurately than any of the other tools tested (Song et al., 2016). Exons overlapping annotated *Alu* repeats in the human genome were identified with BEDtools (Quinlan and Hall, 2010). Lastly, candidate *Alu* exons were selected from among the overlaps with the criteria: i) the exon was internal to the transcript structure, ii) the *Alu* was in antisense to the gene orientation, and iii) the exon was shorter than 400 bp and longer than 40 bp.

### Characterization of Fixed *Alu* Exonization Events

#### Alternative Splicing

We used the tool ASprofile (Florea et al., 2010) to determine alternative splicing events, in particular exon skipping, in the 117 samples. ASprofile compares the exon-intron structures of assembled transcripts to detect patterns indicative of alternative splicing events. Read counts for the introns, representing the number of spliced alignments that contain the intron, were calculated with the tool junc included in the CLASS2 package. Percent Splice In (PSI) values for the intron in each sample were then calculated as PSI = (read_count_iLeft + read_count_iRight)/(read_count_iLeft + read_count_iRight+2*read_count_iSpan)), where iLeft and iRight are the two exons flanking the *Alu* exon in the exon skipping event and iSpan is the exon-skipping intron. Lastly, the median or average of the samples’ PSI values were used in the analysis.

#### Tissue Specific Introns

To determine tissue specificity, we employed the following procedure. We used the Snaptron database and query tool to extract supporting read counts for all introns in the 19,081 samples from 31 tissues in the GTEx database. We used a combination of two tests, to assess: i) the ‘presence’ of the intron in the tissue, and ii) its ‘absence’ from all other tissues. For the ‘presence’ test, an intron is deemed to be ‘present’ in a given tissue if it is present (≥10 reads) in 15% or more of the samples for that ‘tissue’. For the ‘absence’ test, a 2 *× n* matrix (*n* is the number of tissues) is built representing, for each tissue, the number of samples in which the intron has >=10 reads. The first row represents the numbers of samples, for each tissue, from which the intron is expected to be absent, defined as 0.85 * the total number of samples for that tissue in GTEx. The second row contains the observed (O) numbers of samples from which the intron was absent. To perform the test, the matrix is restricted to only those tissues (columns) where O ≥ E, and analyzed with a *χ* ^2^-square test. This test, therefore, excludes any introns that are ‘absent’ at the margin of statistical error. Lastly, introns that were ‘present’ in exactly one tissue (here, brain) and that passed the statistical ‘absence’ test (*p*-value≤0.001) are deemed as tissue specific.

### Detection of Novel (Non-reference) *Alu* Exonization Events

Our approach uses non-concordant paired-end RNA-seq reads since the *Alu*-containing reads will be misaligned in the mapping step. A ‘concordant’ alignment pair is defined as one where the two paired-end reads are mapped to the reference with the correct relative orientations and distance between them. A ‘non-concordant’ read pair is one that is not ‘concordant’, *i.e.* for which there is no concordant pair of alignments anywhere in the genome. When a polymorphic *Alu* is *not* included in the reference genome, the mapped reads containing the unique exonic sequences flanking the exonization event can be used to ‘anchor’ the insertion site. The algorithm follows the following steps: 1) pre-process the data to determine non-concordant reads whose mates match the consensus *Alu* sequence; 2) ‘anchor’ the reads to annotated exons of known genes; 3) determine candidate reads for *Alu* insertions, with the ‘signal’ and ‘shadow’ tests; 4) apply context filters to clusters of reads and determine the likely insertion interval; 5) assemble the reads into contigs; and 6) align the contigs to the gene region to filter likely false positives and select a high confidence set for future validation.

We implemented the algorithm into a software called Alubaster, available without charge under the GPL license from http://github.com/splicebox/Alubaster.

#### Pre-Process the Reads

We classify each read as an *Alu* read or non-*Alu* read using the tool Kraken (Wood and Salzberg, 2014), a fast metagenomic classification tool that matches short sequencing reads to a database of sequences based on their k-mer profiles, and a custom database of *Alu* sequences extracted from the genome-wide *Alu* element annotations. We map all reads to the human genome GRCh38 with Tophat2 v.2.1.1 and extract all non-concordant read pairs in which one mate (say m2) is identified as an *Alu* read. The unique sequence of its mate (m1) will be used to ‘anchor’ the insertion event.

#### Anchor the Reads to Annotated Exons

Determine exons in the GENCODE v.22 annotation overlapping the ‘anchors’, with the program BEDtools (Quinlan and Hall, 2010). Group and then process ‘anchor’ reads by their co-located exons.

#### Determine Candidate Reads for Alu Insertions

One ‘anchor’ exon at a time, process each *Alu*-containing mate to determine whether it is a) a likely ‘signal’, and b) a likely ‘shadow’ match. To gauge potential ‘signals’, the reads are searched against the concatenated sequence of the exon, consensus *Alu* sequence, and the adjacent exon, with a traditional spliced alignment algorithm, sim4db (Walenz and Florea, 2011). The program allows for multiple gaps (insertions, deletions) and substitutions, including longer indels, which may arise when matching the particular instance of the inserted *Alu* against the consensus sequence, and when the insertion site within the *Alu* sequence is unknown. As determined by our studies, a match that explains≥80% of the read sequence, has >80% sequence identity, and covers 10 or more *Alu* bases is deemed a ‘signal’. To determine if the read is likely from a ‘shadow’ local *Alu* element in the genome, it is first searched against a 500 bp intronic region adjacent to the anchor exon (‘unspliced’ test), to eliminate matches due to unprocessed intronic RNA, and then to the genomic region between and including the anchor exon and the farthest adjacent exon (‘region’ test). A match that covers 80% of the read length at 93% sequence identity or higher is deemed real and determined to be a ‘shadow’. *Alu* reads that pass the ‘signal’ filter and are not classified as ‘shadow’ are used to initiate candidate insertion sites at the next step.

#### Apply Context Filters and Infer the Insertion Site

Within each gene, we create a vicinity, or context, around a putative insertion area by clustering overlapping ‘anchor’ reads on the genome. Reads are clustered separately by match orientation and by category, based on the classification of the *Alu* mate, as follows. Specifically, reads (and clusters) that passed the ‘signal’ test above and were not ‘shadows’ are deemed to be strongly indicative of an insertion and classified as S (‘signal’). Reads that tested as ‘shadows’ regardless of the ‘signal’ test are marked with RU (‘regionunspliced’), and deemed likely false positives. To recruit additional potentially informative reads, a hybrid category SN (‘signalnone’) jointly includes S reads and non-‘shadow’ reads that did not pass the ‘signal’ test. Lastly, we created a category SRU (‘signalregionunspliced’) for reads that passed the ‘signal’ test but also tested as ‘shadows’. For each vicinity, we apply additional context based filtering criteria. Let s, sn, ru and sru be the number of reads in the S, SN, RU and SRU clusters. The following criteria are applied to determine an insertion site: i) s >= MIN_SIGNALS; ii) sn >= MIN_SIGNALNONE; iii) the ratio s/sn>=MIN_S2SN; iv) s/sru >=MIN_S2SRU; and v) s/ru >=MIN_S2RU, where MIN_SIGNALS, MIN_SIGNALNONE, MIN_S2SN, MIN_S2SRU and MIN_S2RU are cutoffs that are optimized in an extensive calibration scheme (see below). Conditions i) - iii) are intended to determine that sufficient reads exist to provide evidence for a candidate insertion site, whereas conditions iv) and v) relax the criterion to exclude a site based on the binary presence/absence of ‘shadow’ reads, instead allowing for a proportion of spurious ‘shadows’. To determine suitable parameter cutoffs, we performed an exhaustive calibration and optimization on a simulated data set, as explained below.

Lastly, candidate insertion intervals are determined for each vicinity that passed the context filter above. For each S cluster in the vicinity, an insertion interval is defined as comprised between the initiating S cluster and the closest cluster in the opposing orientation, or the end of the gene if no such cluster exists.

#### Assemble the Reads Into Contigs

We assembled all reads (‘anchors’ and their *Alu* mates) in the clusters bounding the insertion intervals, using the transcript assembler Oases/Velvet (Schulz et al., 2012) with k = 23.

#### Prioritize Contigs

To further select a high-confidence set of candidate inserts, we align all contigs to the full genomic region for the gene, and select those contigs that do not have a high-quality alignment, defined as >=90% sequence identity and >=80% contig coverage. This subset will serve as a starting point for future curation and validation studies.

### Simulation Study and Program Calibration

To calibrate the program and assess its performance, we generated a semi-control data set in which we excised expressed *Alu* elements from the reference genome, and used our method to infer them. We first used HEK293T cell line expression data to infer expressed *Alu* elements, as follows. Reads from one HEK293T RNA-seq sample (SRA Accession: SRR1284895) were mapped to the reference genome GRCh38 and assembled into transcript using CLASS2 v.2.1.6. Exons of the assembled transcripts were then intersected with *Alu* annotations, and 1,000 randomly selected (954 without repetitions) expressed *Alu* elements were excised from the GRCh38 genome to create a new reference genome, GRCh38sim. We also adjusted the coordinates of the GENCODE v.22 gene annotations accordingly. To measure the performance, we ran our algorithm with the SRR1284895 data on the GRCh38sim genome and predicted insertion sites in genes. Note that the data set may contain other real *Alu* element insertions characteristic of the HEK293T cell line, which will be counted as false positives. However, the genome and data set present a realistic and suitable scenario for our goals. For evaluation, we employed standard performance measures, sensitivity Sn = TP/(TP + FN) and precision Pr = TP/(TP + FP), and we used the set of genes harboring deleted *Alu* elements as the gold standard against which to compare the predicted genes.

#### Calibration

To determine suitable cutoff parameters for the context-based filter, in a comprehensive calibration scheme we varied parameters linearly: MIN_SIGNALS = {1, 2}; MIN_SIGNALNONE = {1, .., 10}; MIN_S2SN = {0, 0.1, .., 0.5}; MIN_S2SRU = {0, 0.5, .., 2}, and MIN_S2RU = {0, 0.05, 0.1, 0.15, 0.2, 0.25, 0.5} (see example in Supplementary Figure S2). The maximum Sn that could be achieved was 0.671 and the maximum Pr was 0.8. The best overall performance based on the F-value = 2*Sn*Pr/(Sn + Pr) was obtained for the parameter combination (2,5,0,2,0.5), namely Sn = 291/954 = 0.305, Pr = 291/522 = 0.557, F-value = 0.394, and accuracy Acc=(Sn + Pr)/2 = 0.431, for a run that reported 522 genes, of which 291 were among those listed, *i.e.* true positives (TP), and 231 were ‘false positives’ (FP). We used this set of parameters for our analyses.

## Discussion and Conclusion

*Alu* interspersed repeats represent a large portion of human and other primate genomes, and have played an important part in evolution and potentially the acquisition of species and tissue-specific traits. *Alu* insertions, especially those in gene bodies, have impacted gene structure and function in a variety of ways and at all steps in the regulation of gene expression, including transcription, RNA splicing, RNA editing and translation. *Alu* exonizations, or the recruitment of intronic *Alu* sequences into coding regions by activation of *Alu*-encoded cryptic splice sites, can directly alter gene function and contribute to functional diversification through the formation of alternative gene isoforms. The acquisition of new functions through *Alu* exonization, however, has been a gradual process. The new *Alu* is incorporated in a minority of the gene’s transcripts, where it is able to evolve without significantly impacting the gene’s primary function and therefore under reduced selective pressure (Zarnack et al., 2013; Payer and Burns, 2019). Currently, *Alu* exonizations are estimated to account for ∼5% of alternatively spliced (skipped) exons in the human genome (Sorek et al., 2002). In time, the *Alu* isoform may evolve entirely new function and even become the major isoform for the gene. Hence, the coupling of *Alu* exonization with alternative splicing has provided an elegant mechanism to create functional diversity while safeguarding the primary gene function.

As *Alu* elements continue to insert into the human genome and create structural variants, the process leads to genetic diversity but also potentially to disease. Understanding the prevalence and impact of polymorphic and rare *Alu* insertion variants on the population and in disease at large scale is an important and as of yet little charted endeavor. In particular, when an *Alu* insertion interrupts a gene exon or is very close to an annotated exon, its functional consequence for protein coding capacity of the locus or for mRNA splicing are more likely to be recognized. However, currently there are no adequate tools for characterizing the effects of *Alu* insertions farther from exons in deep intronic space, even when these become incorporated in mRNA transcripts.

Over the past decade, a tremendous volume of publicly available RNA-seq data has been generated and become available, a rich resource that can now be mined to characterize *Alu* exonization events in different tissues, developmental stages and disease conditions. This study taps this potential, as we developed methods to identify exonizations of *Alu* elements with or without *a priori* knowledge of the *Alu* insertion site in the genome, from RNA-seq data. We applied these tools to analyze 117 human frontal cortex RNA-seq samples obtained from the GTEx repository.

Our study detected exonization events at 870 annotated *Alu* elements. In many instances, an *Alu* element was included in multiple *Alu* exons with varying coordinates, to create 1,019 distinct *Alu*-containing exons, and for each *Alu* exon there were potentially multiple flanking introns. Therefore, *Alu* exonization is strongly coupled with alternative splicing. Only 651 of the 1,019 *Alu* exons were uncovered as part of an exon skipping event. There are multiple explanations for this relatively small number. First, exon skipping is defined strictly to refer to exons that are included or excluded alone from gene transcripts, with no changes to the surrounding exons. In multiple examples, including at the UBAP1L and AC103855.2 genes (Supplementary Figure S3), however, we observed multiple exons and/or exon segments being skipped in a single complex alternative splicing event that involves multiple exons or portions of exons, which sometimes include novel unannotated exons (see example at the AC103855.2 gene). Further, some isoforms, particularly those with low expression, may have been missed by the assembler or misassembled. Indeed, a majority of the events had low read counts, with 73% having fewer than 10 reads supporting the flanking introns.

Exonized *Alu* elements belonged to different subfamilies (*Alu*S, *Alu*J and *Alu*Y), suggesting that *Alu* insertions occurring at different times throughout primate and human evolution share a potential to be exonized. Our analyses based on our data set in a single tissue suggest that expression levels measured in read support for the flanking introns are similarly distributed across the three classes, but *Alu*Y exons tend to be included in a lower fraction of a gene’s transcripts compared to *Alu*S and *Alu*J exons, as measured by the PSI value, and are less likely to be in-frame (24.4% compared to 36.2% and 32.8%) (Supplementary Figure S4 and Supplementary Table S4). Collectively these analyses, albeit limited, are consistent with the hypothesis of *Alu* exon birth through exonization and alternative splicing, and evolution to function acquisition. Overall, a majority (∼75%) of all skipped *Alu* exons were present in the minor isoform (PSI<=0.5). When considered alongside the generally low read counts and lack of a preference for in frame exon lengths, these observations suggest that most of the *Alu* exons discovered may play a niche or relatively low impact role in the gene’s function.

*Alu* exons are hypothesized to contribute to species, tissue or condition specific function. Our analyses of 2,771 flanking introns identified 1,260 (45.5%) that were novel, not present in the GENCODE v.36 annotation database. Further, 45 introns (29 novel) were determined to be tissue specific using a strict classification criterion, and a large portion (>21) were in non-coding RNA genes.

In contrast to fixed *Alu* exonizations, which are common in the population and may have been evolutionary selected, polymorphic or rare *Alu* insertions occur in a small number of samples and are more likely to be present at a low expression level in a carrying individual. These characteristics alone make them difficult to detect from the transcriptomic data. Further, there are significant computational challenges to detecting gene *Alu* insertions at uncharted non-reference locations and from whole-transcriptome RNA sequencing data, where an *Alu* read may match to thousands of genomic locations. While paired-end reads can help localize the search, local *Alu* elements located in the gene’s introns and exons present additional sources for the exon, leading to false positives. Consequently, despite the great interest in their potential to point to the mechanism and cause of uncharacterized genetic diseases, polymorphic *Alu* insertions giving rise to exonizations may be underreported by our pipeline and even more so in disease databases.

In conclusion, our novel software Alubaster detected putative *Alu*-containing exons in hundreds of genes expressed in the human cortex, with over half seen in multiple samples. These events can be leveraged on their own, or can be incorporated into gene annotations and other feature (exon, intron) databases that can be queried or included in differential splicing analyses, to allow the discovery of novel markers of disease. While further manual curation and experimental validation will be needed to confirm and characterize each event individually, we believe that our collection of tools, sites and sequences represents a valuable resource that can be employed to understand the characteristics of *Alu* (m)RNA insertions.

## Data Availability

The original contributions presented in the study are included in the article/Supplementary Material, further inquiries can be directed to the corresponding author.
