# Supplementary material for: Detection of Alu Exonization Events in Human Frontal Cortex From RNA-Seq Data
Source: Front Mol Biosci. 2021 Sep 10;8:727537. doi: 10.3389/fmolb.2021.727537 (PMC8460874; doi:10.3389/fmolb.2021.727537)
Supplement: Supplementary file 1 [file DataSheet1.docx]

SUPPLEMENT TO THE PAPER

“DETECTION OF ALU EXONIZATION EVENTS IN HUMAN FRONTAL CORTEX FROM RNA-SEQ DATA”

L. FLOREA, L. PAYER, C. ANTONESCU, G. YANG and K. BURNS

**Table of Contents**

**Supplementary Figure S1.** PSI values of fixed *Alu* exonization events.

**Supplementary Figure S2.** Calibration study (excerpt) for parameter optimization.

**Supplementary Figure S3.** Examples of exonization events at fixed (in the reference genome) *Alu* elements.

**Supplementary Figure S4**. Histograms of (A) readcounts, (B) PSI values, and (C) coding frame distribution for the *Alu*J, *Alu*S and *Alu*Y classes.

**Supplementary Figure S1.** PSI values of fixed *Alu* exonization events. Percent Splice In (PSI) values of exon skipping (ES) events of *Alu* exons were computed per sample, then a unified value (mean or median, respectively) was calculated across the samples and plotted into a histogram. (A) Calibration: mean versus median correlation for *all* Alu exon skipping events (left), and for events with >=10 reads only (right). (B) Histogram of PSI values (mean across samples) for *Alu* events when only samples with >=10 reads were considered, by comparison to all exon skipping events in the 117 sample data set (39,859 exon skipping events with >=10 reads).

(A)

(B)

**
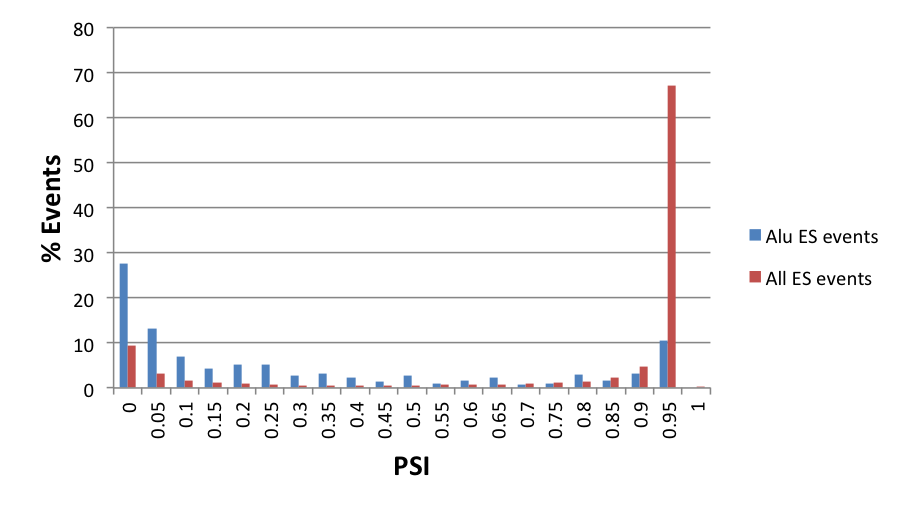
**

**Supplementary Figure S2.** Calibration study (excerpt) for parameter optimization. Program parameters were varied across the following ranges: MIN_SIGNALS = {1,2}; MIN_SIGNALNONE={1,.., 10}; MIN_S2SN={0, 0.1, .., 0.5}; MIN_S2SRU={0, 0.5, .., 2}; and MIN_S2RU= {0,0.05,0.1,0.15,0.2,0.25,0.5}. For each combination, Sn=TP/(TP+TN) and Pr=TP/(TP+FP) values were computed and represented in a scatterplot, with Sn represented along the horizontal axis and Pr along the vertical axis. The minimum overlap with the consensus *Alu* element, and the fraction of the read covered by the alignment in the ‘signal’ test, were set at r=10nt and c=0.8, respectively, for the experiment shown.

**
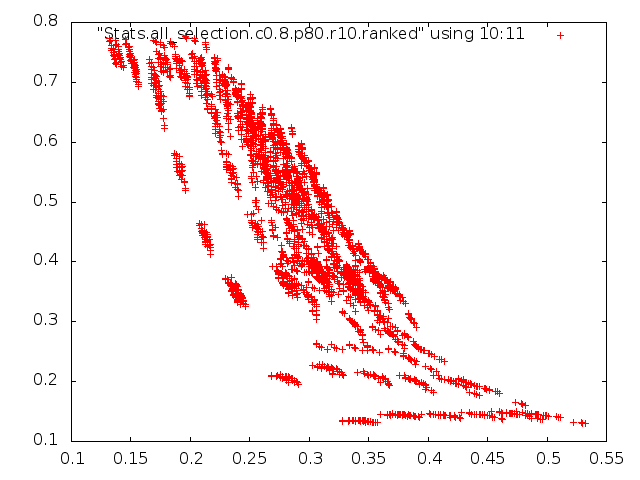
**

**Supplementary Figure S3.** Examples of exonization events at fixed *Alu* elements. (A) A gain of 5’ss at a 3’ UTR terminal exon at the PDZD7 gene creates a 363 bp internal *Alu* exon. The exon region is highlighted in blue on the display. The gene is primarily expressed in cortex, and there is no sequence conservation outside of the primate region for the *Alu* portion of the exon. (B) New *Alu* exon at the UB1APL gene. The downstream intron is tissue specific and not found in the GENCODE v.36 database. (C) A new, brain-specific *Alu* exon, and multiple novel (unannotated) splicing patterns at the non-coding RNA gene AC103855.2. (D) GENCODE confirmed *Alu* exonization at the brain-specific GABRG2 gene. Displays were generated with the Integrative Genomics Viewer and the UCSC Genome Browser.

(A)

(B)


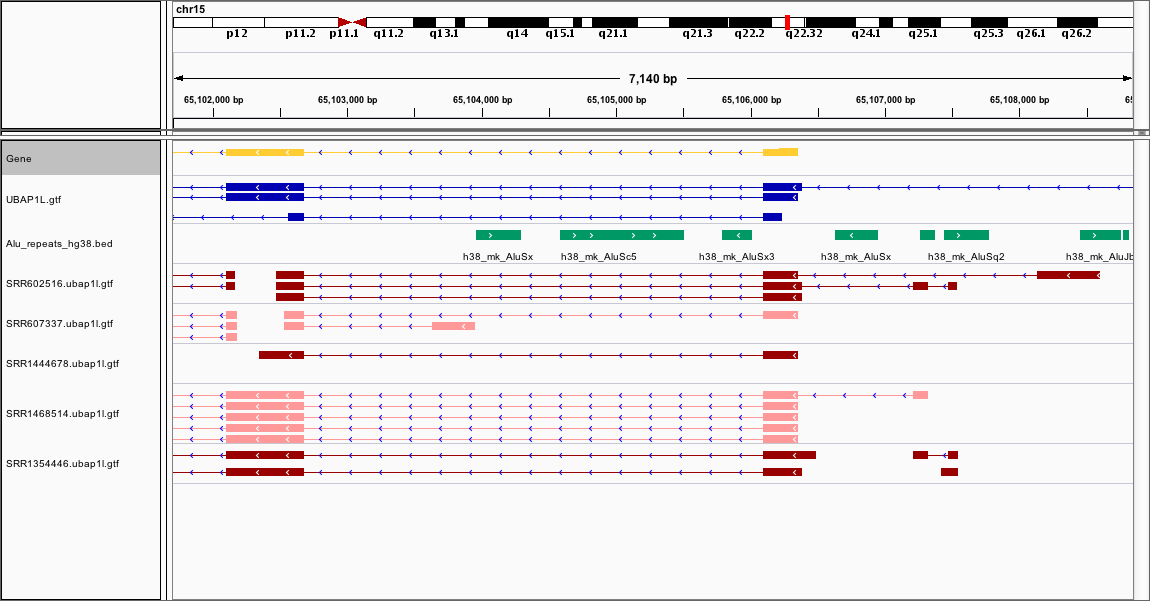


UBAP1L

(C)


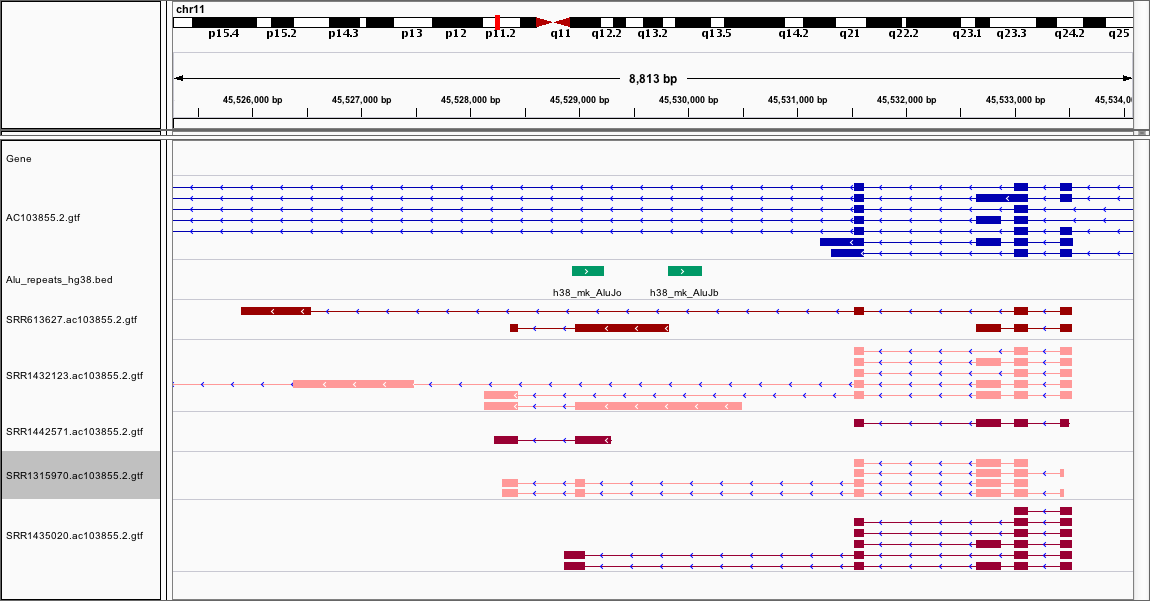


AC103855.2

(D)

**Supplementary Figure S4**. Histograms of (A) read counts, (B) PSI values, and (C) coding ‘frame’ (exon length mod 3) distribution for the *Alu*J, *Alu*S and *Alu*Y classes.

(A)


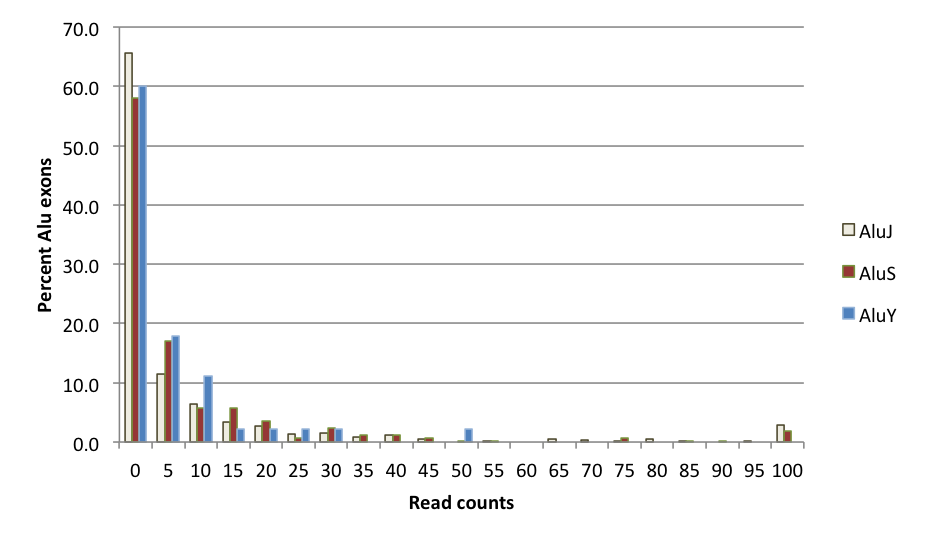


(B)

(C)
